# Supplementary material for: Association of Serum High-Density Lipoprotein Cholesterol with High Blood Pressures at Checkup: Results of Kanagawa Investigation of Total Checkup Data from the National Database-9 (KITCHEN-9)
Source: J Clin Med. 2021 Oct 30;10(21):5118. doi: 10.3390/jcm10215118 (PMC8584897; doi:10.3390/jcm10215118)
Supplement: Supplementary file 1 [file jcm-10-05118-s001.zip › jcm-1408030-supplementary.pdf]

Whole subjects

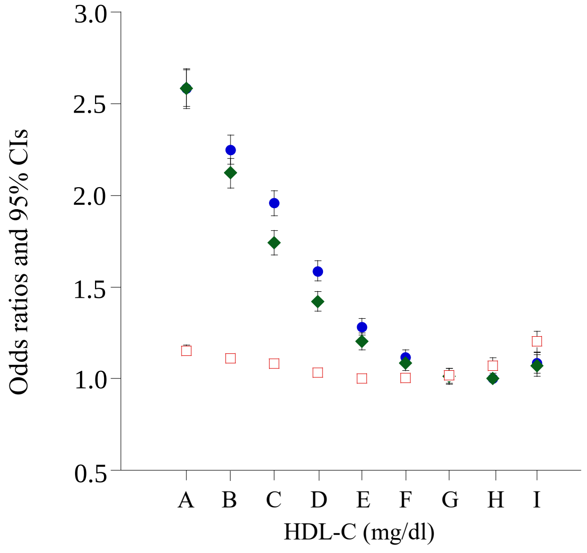

Men

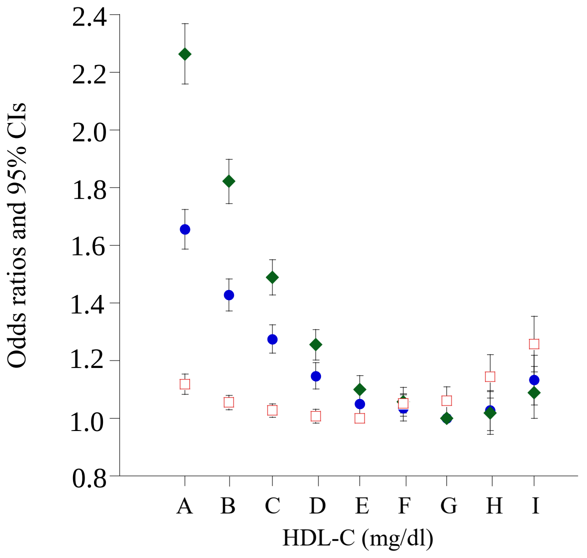

Women

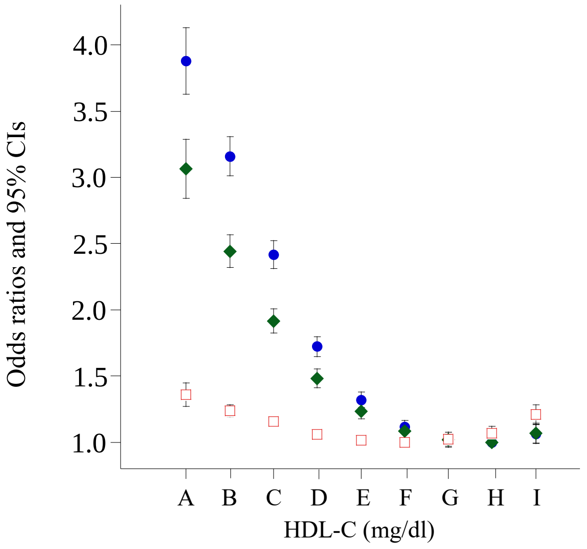

Figure S1: Odds ratios of nine HDL-C concentration categories for pharmacotherapy for hyper-tension.
